# Sociolinguistic monitoring and L2 speakers of English

**DOI:** 10.1515/ling-2023-0073

**Published:** 2024-09-20

**Authors:** Jana Pflaeging, Bradley Mackay, Erik Schleef

**Affiliations:** Department of English and American Studies, 27257 University of Salzburg, Salzburg, Austria; Freiburg Institute for Advanced Studies (FRIAS), 27257 University of Freiburg, Albertstraße 19, 79104 Freiburg im Breisgau, Germany

**Keywords:** sociolinguistic monitoring, L2 variation, sociolinguistic variation, proficiency-based variation, social meaning

## Abstract

This study contributes to a growing body of research on the social meanings of linguistic variation with particular interest in the cognitive processes governing their emergence. Our research follows in the tradition of Labov et al.’s (2011) work on the sociolinguistic monitor, a cognitive mechanism hypothesized to track quantitative linguistic variation and prompt social evaluations (Labov et al. 2011. Properties of the sociolinguistic monitor. *Journal of Sociolinguistics* 15(4). 431–463). Previous research shows that L1 English listeners are sensitive to frequency variation, but it is unclear whether this also applies to L2 listeners. This study thus replicates Labov et al.’s (2011) original experiment in a context where English is primarily acquired through L2 instruction. To test the generality of *sociolinguistic monitoring*, we investigate L2 listeners’ sensitivity to quantitative differences in sociolinguistic variation (ing) as well as proficiency-based variation. Since participants were L1 speakers of (Austrian) German, we tested evaluations of varying realizations of /θ/ ([θ]/[s]), /d/ ([d]/[t]), and /w/ ([w]/[v]). Experiments included 135 participants, who rated several versions of newscaster test passages regarding *professionalism*. Our data shows that both sociolinguistic and proficiency-based variation are monitored and evaluated by L2 listeners, albeit to different extents. This supports the assumption that the focus of the monitoring process is *socially meaningful variation* that includes L1 sociolinguistic but also L2 proficiency-based features.

## 1 Introduction

Recent work in variationist sociolinguistics, undertaken in the “third wave” of variation studies (Eckert 2005), has devoted much attention to the question of how linguistic forms are tied to social meanings, that is, evaluations and stances, personal traits, social personae, or types (Moore and Podesva 2009: 448–450). Just what meaning(s) of a variable, or rather its respective variants, are triggered in spoken interaction is highly dependent on a range of factors, including listeners’ expectations, prior experiences, beliefs, attitudes, and various contextual conditions (Schleef and Flynn 2015: 50; also Stecker 2020: 119). When conceptualizing the indexical relationships between linguistic forms and their social meaning(s), scholars have also emphasized the importance of *cognition* (e.g., Campbell-Kibler 2009: 136–137, 2011: 423; Labov et al. 2006, 2011; Levon 2014; Wagner and Hesson 2014: 645). Social meanings are essentially “social content tied in the minds of a given speaker/hearer to a particular piece of linguistic behavior” (Campbell-Kibler 2009: 136). The construction of meaning based on linguistic variation is also said to be impacted by *social salience*, that is, sociocognitive processing constraints that can “moderate the amount of attention that listeners devote to perceiving a speech signal” (Levon 2014: 560; see also Levon and Fox 2014: 185). While the level of engagement with cognitive aspects in third-wave research has been described as low (Campbell-Kibler 2011: 424), Labov et al.’s (2006, 2011) work on sociolinguistic monitoring has developed into an experimental paradigm that a number of scholars have since contributed to.

Building on the results of several experimental studies, Labov et al. (2006, 2011) conceptualize the *sociolinguistic monitor* as a cognitive mechanism that processes and tracks quantitative linguistic variation in real time. Based on remembered tokens and patterns, varying frequencies of linguistic variants were found to give rise to (degrees of) social meaning (Labov et al. 2011; also Levon and Fox 2014: 186). Labov et al. argue that this tracking may be carried out by “a separate processing and storage *module*” (Labov et al. 2011: 434, our emphasis) or it may be done “‘on the fly’ at any time by an inspection of remembered tokens”. Labov et al. (2011: 434) are “neutral to this issue” and our study was conducted in the same spirit. To avoid equating *monitor* with *module*, we refer to the notion of *sociolinguistic monitoring*, shifting the focus to the process of monitoring variation.

Results from replication studies have tested the generality of sociolinguistic monitoring by exploring the evaluation of frequency variation in relation to certain language-external and -internal factors, e.g., regional background (Labov et al. 2006, 2011), gender/sex (Stecker 2020), pragmatic language ability (Wagner and Hesson 2014), a variable’s salience (Levon and Fox 2014), phonological context (Freitag 2020; Labov et al. 2006, 2011), and structural level (Levon and Buchstaller 2015), as well as word frequency/typicality (Vaughn 2022). The question of whether L2 listeners show a similar sensitivity to quantitative variation of linguistic forms, however, has remained largely unexplored. A possible response pattern is described in Labov et al. (2011: 445), who argue that “the sociolinguistic evaluation of (ing) is quite general. […] non-native fluent speakers of English responded much in the same way as native speakers”. As their experiments included only six fluent L2 listeners, further empirical research is likely to generate more robust results. Thus, it is one of our aims here to explore whether the frequency of [ɪn] is monitored and evaluated by L2 listeners, who learned English as a second (or other) language, in the same way as Labov et al.’s results suggest. In addition, we seek to investigate how specialized the process of sociolinguistic monitoring is. While previous research within the paradigm has focused on *sociolinguistic* variation, other types of variation might be governed by the same processing and tracking mechanisms. Linguistic variation attributable to a particular L2 accent is particularly suitable for such research as it is also associated with positive or negative evaluation, yet this variation is not – strictly speaking – sociolinguistic. Consequently, the present paper has two main goals:

1. To replicate Labov et al.’s original study with more data and several test passages in a context in which English is primarily acquired through L2 language instruction.
2. To test the generality of sociolinguistic monitoring to features that are not (strictly speaking) sociolinguistic but result from proficiency-based variation.

We proceed by, firstly, describing the original experiment by Labov et al. and by providing a brief summary of replication studies that have been conducted in recent years. Based on previous findings, we identify some established features of sociolinguistic monitoring, and point to contradicting results and open questions. Secondly, to provide further context to our own empirical study, we survey previous research on (ing) and associated patterns of sociolinguistic variation. This is then contrasted with the phenomenon of proficiency-based variation, focusing particularly on /θ/ as [s] (ths), final devoicing of /d/ as [t] (dt), and /w/ realized as [v] (wv), which are relevant to (Austrian-)German learners of English. Thirdly, we outline the perception experiments that we conducted, introduce our results and assess them to conclude the paper.

## 2 Background

### 2.1 Sociolinguistic monitoring: previous studies

#### 2.1.1 Original experiment

The existence of a *sociolinguistic monitor* was first postulated just over 15 years ago, when Labov et al. (2006, 2011) sought to further investigate listeners’ sensitivity to fine-grained quantitative differences in the production of linguistic variants and the effect of frequency variation on social evaluations. As part of the original experiments, the social context of a news broadcast was selected (Labov et al. 2006: 109; Labov et al. 2011: 434). This context has been shown to prime overt, prestigious social norms in relation to standard features (e.g., Labov 1966; also Labov et al. 2011: 434), which allows for eliciting evaluations of speaker competence or professionalism. Stimuli were constructed on the basis of a written news broadcast, which featured ten progressive {*-ing*}-suffixes across seven sentences. The text was recorded with a female speaker of standard American English, who varied her articulation of (ing) between [ɪŋ] and [ɪn] (Labov et al. 2011: 436–437). In a slightly adapted version of the initial experiment, Labov et al. created an experiment with only seven guises, which differed in the number of marked velar (ing) realizations ([ɪŋ]) used to replace the original alveolar (ing) variants ([ɪn]). In particular, one guise was left unaltered and showed [ɪŋ] throughout (0 %); others included one (10 %), two (20 %), three (30 %), five (50 %), seven (70 %), and all instances (100 %) of [ɪŋ] replaced with [ɪn] (Labov et al. 2006: 109; Labov et al. 2011: 436–437).

The experiment was administered to 36 L1 speakers of American English (e.g., Labov et al. 2011: 441), who were informed that they would hear seven test takes of a newscast by a journalism student to be handed in with an application to a local radio station. They were instructed to judge each recording on a rating-scale, ranging from *perfectly professional* (1) to *try some other line of work* (7). Results indicated that listeners were indeed able to perceive relatively subtle differences in frequency with which the speaker produced alveolar [ɪn]-variants. Recordings with more [ɪŋ]-articulations were evaluated as more professional; versions with more [ɪn]-variants, in turn, were regarded as less suitable for the job as a newscaster. Labov et al. found that almost 40 % of listeners’ ratings follow a logarithmic rather than a linear progression (e.g., Labov et al. 2011: 439, 442): while low [ɪn]-frequencies triggered relatively sharp shifts in evaluations, guises with higher [ɪn]-frequencies showed a ceiling effect, with smaller differences between ratings from the 50 %-guise onward (see also Stecker 2020: 120).

#### 2.1.2 Replications and other related studies

In order to explore this effect further, Labov et al. replicated and adapted their original study, e.g., by letting participants express finer distinctions in their evaluations in real time, by re-running experiments in different locales and by testing for an interaction with other dialect features (see Labov et al. 2006: 115, 117–124; Labov et al. 2011: 448–449). Experiments yielded a similar logarithmic fit and further evidence that L1 subjects are monitoring and responding to occurrences of the alveolar variant [ɪn] (Labov et al. 2011: 456). They also found that listeners from South Carolina evaluated the production of [ɪn] less stringently (Labov et al. 2011: 447) but generally followed the logarithmic progression previously described; the co-presence of marked southern dialect features had no further effect on evaluations (Labov et al. 2011: 449). As this high degree of overlap between results was not quite expected, Labov et al. called for “further trials with less salient sociolinguistic variables […] to determine the generality of the logarithmic response pattern” (Labov et al. 2011: 457).

In response to this call, Levon and Fox (2014) replicated Labov et al.’s original experiment in the UK, where (ing) variation, they argue, attracts less evaluative attention and can thus be assumed to have lower social prominence or salience than in the US (Levon and Fox 2014: 186–187, but see Schleef et al. Under review a). Their findings were in line with these assumptions, as listeners displayed no sensitivity to quantitative variation of [ɪn] in London. There was no evidence of a logarithmic distribution; instead, average professionalism ratings indicated a flat, linear distribution of evaluations across the stimuli and a weak attitude strength overall (Levon and Fox 2014: 197–198). A follow-up experiment on the perception of (th)-fronting, a variable expected to attract stronger evaluations in the UK (Levon and Fox 2014: 201), yielded a similar lack of sensitivity to different [f]-frequencies (Levon and Fox 2014: 204). Upon closer scrutiny, however, significant differences between participant groups indicated that northern listeners exhibit more negative attitudes toward [f] for /θ/ than southerners. This effect was hypothesized to result from a higher perceptual sensitivity due to attitude strength among northern listeners to a feature that is still viewed as emblematic of southern British English (Levon and Fox 2014: 207).

Developing the experimental paradigm further, Wagner and Hesson (2014) explored inter-individual variation in the perception of different (ing)-variants among L1 speakers of US English as induced by differences in (emotional) intelligence, personality traits, or ‘cognitive style’ (see Ausburn and Ausburn 1978, qtd. in Wagner and Hesson 2014: 655).1In reference to research in psychology (Ausburn and Ausburn 1978), Wagner and Hesson conceptualize *cognitive style* as an “individual’s […] particular manner of attending to and processing information” (Wagner and Hesson 2014: 655). To achieve a systematic assessment of relevant cognitive characteristics, they implemented the *Broad Autism Phenotype Questionnaire*. It allows to determine, for instance, whether individuals have a preference for patterns and repetitions, which poses a direct conceptual link to linguistic frequency variation and meaning attributions. Wagner and Hesson (2014: 656) used the same audio files as Labov et al. (2011) and found that, at least for the middle range of [ɪn]-frequencies, comparably better pragmatic language skills were a predictor of lower professionalism ratings as the use of the marked variant increases (Wagner and Hesson 2014: 661). Results show that their data is best explained by a logarithmic model, although it accounts for a smaller proportion of the variation when compared with findings from Labov et al. (2011). Levon and Buchstaller (2015) compared the perceptual dimension of quantitative sociolinguistic variation of a phonological variable, (th)-fronting, with that of a morpho-syntactic one, the use of {*-s*} for subject NPs other than 3SG (see Levon and Buchstaller 2015: 321, 324). Their results suggest that grammatical variation may also give rise to social evaluations, although in a more complex fashion since judgments were contigent on listener region as well as respondents’ pragmatic language ability (Levon and Buchstaller 2015: 336–337). The effects Levon and Buchstaller found (e.g., frequency of (th)-fronting or Northern-Subject Rule on perceived professionalism) were linear, not logarithmic (Levon and Buchstaller 2015: 333, 335). Freitag (2020) looked into L1 Portuguese listeners’ evaluations of two types of palatalization processes as triggered by different phonological environments. It needs to be kept in mind that the guises employed as part of her study were noticeably shorter than those used by Labov et al. However, her findings further support the assumption that quantitative sociolinguistic variation is monitored by L1 speakers and indicate a curvilinear pattern for some of the phonological contexts investigated (Freitag 2020: 26). She also identifies listeners’ reaction time as a promising parameter to consider in future research (Freitag 2020: 29).

A recent study by Stecker (2020) explores how far a speaker’s perceived gender identity influences listeners’ ratings of [ɪn]-frequencies with regard to professionalism as well as other social attributes, such as likability, intelligence, and likelihood of being a real-life newscaster (Stecker 2020: 124). Unlike previous studies, guises covered all quantities from zero to ten tokens (except a 90%-guise) and were recorded with ten different L1 speakers of US English, yielding 100 stimuli in total (Stecker 2020: 121). The study involved 186 participants, who heard a randomly ordered sample of ten stimuli each, incl. all speakers and [ɪn]-frequencies. Except for evaluations regarding likability, which were not found to be significantly predicted by [ɪn]-frequency (Stecker 2020: 123), results for all other attributes were in line with many findings by Labov et al. (2006, 2011): [ɪn]-frequency was found to be a significant predictor of overall ratings regarding professionalism, intelligence, and likelihood of being a real-life newscaster. Unlike findings from Labov et al., the data was best described by a linear rather than a log-transformed regression (Stecker 2020: 123, 125). Similar to previous findings, speaker gender did not lead to significant differences between ratings for the social attributes under scrutiny (Stecker 2020: 125).

Finally, Vaughn (2022) investigated whether listeners’ expectations regarding a word’s *typical* grammatical use (i.e., the word ending in {*-ing*} is most frequently used as a noun vs. verb, e.g., *building*) or a word’s *local* grammatical use (i.e., use as a noun or verb in a given test sentence) have an impact on the social evaluation of different (ing)-variants. Based on ratings of five versions of a newscast passage on a seven-point *professionalism*-scale, Vaughn found that it is a word’s *typical* (i.e., most frequent) grammatical form that gives rise to social evaluations rather than a word’s use in a given sentence. With typical nouns, fewer tokens of [ɪn] were sufficient to make a guise sound less professional to listeners, compared to {*-ing*}-words that are typically verbs. When plotted in a graph, the response pattern appeared to follow a logarithmic distribution rather than a linear one for typical nouns, but not for typical verbs. However, these assumptions were not verified through statistical testing (Vaughn 2022: 515–516).

#### 2.1.3 Implications and open questions

The partial overlap between response patterns found in the original experiments, and the fact that the similarity of results could not be explained by any previous findings on (ing), led Labov et al. to conclude that their findings “must depend on some fundamental property of speech perception, perhaps specific to sociolinguistic monitoring, perhaps based on more general properties of perception” (2011: 451). They postulate the existence of a sociolinguistic monitor, that is, a cognitive mechanism that processes and tracks quantitative linguistic variation in real time and prompts the attribution of social meanings based on remembered tokens and patterns (Labov et al. 2011: 434–435; also Levon and Fox 2014: 186). This also implies that sociolinguistic perception is not categorical but gradual in nature (Levon and Fox 2014: 186). As suggested above, results from replication studies seem to provide further support for these assumptions. At the same time, they challenge some of the claims which have been made and pose further questions.

In view of previous research, the sociolinguistic monitor – or rather, sociolinguistic monitoring – can be characterized as follows:

– **Temporal range**: Labov et al. (2006, 2011: 457) found that the temporal window of the monitor(ing) extends at least to 57 s. Unfortunately, many other studies refrain from making statements about the length of the stimuli used.
– **Sensitivity**: With regard to frequency distinctions, Labov et al. (2006, 2011) found a high degree of consistency with adult listeners being able to track differences as small as 10 %. Levon and Fox’s (2014) study, however, called the general applicability of this characteristic into question. Their results suggest that sensitivity also depends on the degree of social salience of the variable. Wagner and Hesson’s findings suggest that the sensitivity of the monitoring mechanism “may be more related to impression formation than attention, as it seems to mediate the extrapolation of social attitudes from relatively subtle patterns in linguistic data” (2014: 661–662).
– **Attenuation**: With regard to professionalism ratings, replications by Labov et al. (2006, 2011) have provided further support for the ceiling effect that results from minor differences between evaluations beyond the 50 %-guise. However, several other studies (e.g., Freitag [2020]; Levon and Buchstaller [2015]; Levon and Fox [2014]; Stecker [2020]; and also Vaughn [2022: 517], though she did not compare the models statistically) suggest that, in some cases, data might be better described by a linear rather than a log-transformed regression. This means that, instead of a reduced effect of every consecutive token that is indicated by a logarithmic progression, the linear response pattern suggests every token has the same effect on listeners’ evaluations.
– **Structure**: Levon and Buchstaller’s (2015: 319–320) results refute earlier claims that the monitoring mechanism is only sensitive to surface forms and suggest that it equally applies to higher-level grammatical variables, although responses were found to be conditioned by a number of factors.
– **Gender/sex**: As with Labov et al. (2006, 2011: 442–443), follow-up studies that investigated gender effects on professionalism ratings (and others) have reported no significant differences between male and female respondents (see Levon and Buchstaller 2015: 331; Levon and Fox 2014: 191; Stecker 2020: 125; Wagner and Hesson 2014: 659).
– **Age of acquisition**: Labov et al. (2011: 452–454) also reported differences between response patterns of adolescents versus adult respondents. Their discussion of results, however, has attracted substantial criticism (see Levon and Fox 2014; also Freitag 2020: 23). Levon and Fox (2014: 192–193), for instance, point out that alternative interpretations of the data are possible and need to be related to age-group-specific degrees of social status and the salience that a variable may have.2In particular, the lack of a logarithmic response pattern among adolescents could result from a generally lower social status (and hence salience) of [ɪŋ] for younger listeners.
– **Range of social attributes**: Results from replication studies indicate that sociolinguistic monitoring equally applies to social attributes other than professionalism (see e.g., Stecker 2020).
– **Individual differences**: Wagner and Hesson’s (2014: 662) study raised awareness of inter-individual differences between respondents that led to diverging evaluations for particular mid-level guises.

The growing body of work on sociolinguistic monitoring has established an experimental paradigm that provides a rich context for further research into potential factors that condition listeners’ ratings of socially salient quantitative variation in language use. Many of the studies mentioned above outline several avenues for future research. Acknowledging the importance of these tasks, we wish to shift into focus yet another aspect that seems worthy of further scrutiny: the workings of sociolinguistic monitoring in contexts where English is acquired as a second (or other) language. Discussing results from their initial experiments, Labov et al. claim that “non-native fluent speakers of English responded much in the same way as native speakers” (2011: 445). As their sample consists of six L2 participants only, further experimental work is needed to draw more robust conclusions (Labov et al. 2011: 445). In the following, we thus turn to questions of sociolinguistic and proficiency-based variation as produced, perceived, and evaluated by (Austrian-)German L2 speakers of English. Before we do this, we provide some important background information on the social meanings of (ing) and then extend the discussion to proficiency-based variation.

### 2.2 Evaluation of sociolinguistic and proficiency-based variation

#### 2.2.1 Sociolinguistic variation: the variable (ing)

The study of sociolinguistic variation accounts for the relationships between observable variation in language use, social parameters such as class, ethnicity, gender, social networks, ideology or identity, as well as individual styles (Eckert and Rickford 2001: 1–2). Empirical research has traditionally focused on particular sociolinguistic variables, with (ing) being one of the most commonly examined features (Campbell-Kibler 2007: 35). For the US context, it has been shown that there is systematic alternation between velar ([ɪŋ]) and alveolar realizations ([ɪn]) of the unstressed verbal suffix {*-ing*}. Thus, the progressive form *singing* can either be pronounced as [ˈsɪŋɪŋ], or as [ˈsɪŋɪn] (or [ˈsɪŋən], see Campbell-Kibler [2007]: 32). The use of (ing) has been described as highly stable, with regular social and stylistic stratification across a range of English varieties (Hazen 2006; Houston 1985; Labov 2001; also Campbell-Kibler 2007: 35). The alveolar variant [ɪn] exhibits a tendency to be used somewhat more frequently by male speakers and increases in frequency as speech styles become more informal (Hazen 2006; Labov 2001; also Schleef et al. 2017: 33). Perception studies have suggested that (ing) is also a comparably salient variable, whose variants tend to attract a number of social evaluations. Using the matched-guise technique, Campbell-Kibler found that US speakers who produced [ɪŋ] were, for instance, rated as significantly more educated (Campbell-Kibler 2007: 47), intelligent, articulated, and as more likely to be a student, from the city and “gay-sounding” (Campbell-Kibler 2007: 32–33, 55; Campbell-Kibler 2011: 423). [ɪn]-guises, in turn, tend to prompt associations with informality, uneducatedness, and the country (Campbell-Kibler 2007: 33, 49; Campbell-Kibler 2011: 423). As elaborated above (see Labov et al. 2006, 2011), listeners also show a sensitivity to quantitative differences in the use of (ing)-variants. Moreover, (ing) has also been the subject of explicit meta-commentary, which suggests that it has made its way into the conscious awareness of US speakers (Campbell-Kibler 2007: 55).

Compared with the US context, the social and stylistic stratification of (ing) is similar in the UK. This is especially true for southern varieties of British English where the [ɪŋ]-variant is the prestigious form and tends to be used by MC and UC speakers (Levon and Fox 2014: 195). For a number of socio-historical reasons, however, the use of (ing) is notably less socially conditioned in the north of England and Scotland (Schleef and Flynn 2015: 52; Schleef et al. 2017: 32–33, in ref. to Tagliamonte 2004: 401; also Levon and Fox 2014: 195). Research into the social meanings of (ing) has revealed that, independent of region, MC listeners agree on the formality of [ɪŋ] (Schleef et al. 2017: 46). However, WC listeners in different locales may hold different attitudes for some social-status scales. As mentioned above, the evaluation of [ɪn] has also been reported to be less negative in London when compared to results of Labov et al. (Levon and Fox 2014, but see Schleef et al. Under review b).

Our review suggests that a variety of factors impact the evaluation of (ing). However, there is still much unexplored terrain when it comes to perceptual aspects of sociolinguistic variables, even if they have been studied as extensively as (ing). For instance, to what extent L2 speakers of English acquire the range of social meanings associated with (ing) has so far received only minor attention. It is one of the objectives of the present study to address and expand the L2 research initiated by Labov et al. (2011) and scrutinize data on how (Austrian-)German L2 speakers of English perceive quantitative variation of (ing) as used by an L1 and an L2 speaker of English. Experiments on the perception of sociolinguistic variation are complemented with those that focus on proficiency-based variation.

#### 2.2.2 Proficiency-based variation: (THS), final devoicing (DT) and (WV)

Apart from instances of sociolinguistic variation, language use may also show variation induced by different levels of foreign language proficiency, which we shall refer to as *proficiency-based variation*. It is understood here as cases where speakers produce linguistic forms that may appear deviant and, hence, more or less acceptable when compared to a standard British or American L1 model. While proficiency-based variation can occur on all levels of linguistic description, it is standard L1-like pronunciation that tends to pose challenges even to advanced learners, who otherwise have no difficulty producing lexically rich and complex syntactic structures (see Beinhoff 2013: 36–37).

Variation in L2 pronunciation has been argued to result from various types of sound transfer, such as *sound substitution*, that is, cases where an L2 learner falls back on the closest L1 equivalent (Weinreich 1953, qtd. in Major 2008: 67). The acquisition of L2 phonology may also involve other types of sound interferences that arise from *underdifferentiation*, a situation where sounds are allophones in the L1 but separate phonemes in the L2 (Weinreich 1953, qtd. in Major 2008: 77). These and other transfer phenomena are attestable in L2 English spoken with a German accent, although the extent to which they occur varies with level of proficiency. Typical accent features of German L2 English speakers include the articulation of /θ/ as [s], the devoicing of word-final /d/ as [t] in all contexts, and the replacement of word-initial /w/ with [v] (Beinhoff 2013: 49, 51; see also Mair 1995; Smith et al. 2009: 257; Wieden and Nemser 1991).

Research has found that L2 sounds that show similarity to those present in an L1 are harder to acquire since the smaller the differences are, the more likely it is that they go unnoticed (Major 2008: 72). Also, in cases where transfer and developmental processes in the L1 overlap and result in the same substitution, the substitution has been shown to persist longer (Major 2008: 76). Both cases seem to apply to German learners of English (see König and Gast [2018, Chapter 2] for a contrastive analysis of the phoneme inventories of English and German). /θ/ and /w/, for instance, are not part of the phoneme inventory of German and, thus, sound-substitution phenomena are likely. The acquisition of the interdental fricative, for example, can be expected to pose some difficulty for German learners due to a perceived similarity in the manner and place of articulation between English /θ/ and German /s/ (Beinhoff 2013: 60). Likewise, German learners of English may articulate /w/ as [v] due to the fact that both are labial sounds, thus [v] may seem the closest L1 equivalent. In addition, there is a regular correspondence between the German grapheme <w> and /v/ in pronunciation. Finally, the phenomenon of final devoicing (resulting from *underdifferentiation*) can be expected to persist even longer as it is also influenced by developmental processes in German L1 acquisition (Major 2008: 76; see Smith et al. 2009: 257). In conclusion, we are investigating two transfer features associated with English acquisition among German speakers ([s] for /θ/ and [v] for /w/) as well as one transfer feature (final devoicing) that still occurs in more advanced learners of English.

The question of whether a learner’s spoken output is regarded as acceptable is deeply intertwined with social evaluations and attitudes (Beinhoff 2013: 38). Such evaluations – be they implicit or explicit (Petty et al. 2009, qtd. in McKenzie 2015: 34) – are typically cultivated in contexts of foreign-language teaching and learning, where curricula and assessments are based on an L1 standard (Beinhoff 2013: 47; Lindemann et al. 2014: 171, 179). This leads to a situation where compliance with standard L1 features implies ‘correctness’ and ‘educatedness’ (Beinhoff 2013: 47) and a deviation from the norm is regarded a production error. This is especially so if the speaker is believed to be an L2 speaker of the language in question, as empirical studies have shown (Lindemann 2017: 198–199).

Although research into attitudes toward L2 accents is still comparably rare (Beinhoff 2013: 25, 29), several studies have shown that language learners tend to internalize the stigma typically associated with L2 varieties (Lindemann et al. 2014: 176, 178; see also Beinhoff 2013: 31; Dalton-Puffer et al. 1997: 121).3A more recent line of research at the intersection of language, race, and the work domain has uncovered that such internalized stigma likely result in inherently racist pedagogical initiatives, e.g., accent reduction programs for migrant workers (Ramjattan 2023: 39). Given the often extensive period of time over which L2 learners experience L2 instruction and assessment in school education and, at times, at university as well (see e.g., Dalton-Puffer et al. 1997: 115–116), it is perhaps not surprising that L2 learners exhibit negative attitudes toward their own in-group L2 accents (Beinhoff 2013: 32; Lindemann et al. 2014: 180). The attitudes that German and Austrian learners of English have toward their own L2 accents is a case in point (Dalton-Puffer et al. 1997: 121, 125–126; also Teufel 1995). When L2 English learners from Austria were asked to rate L1- and L2-accented versions of a radio announcement, participants showed clear preference for the L1 English standard pronunciation (Dalton-Puffer et al. 1997: 125). Austrian accents in American and British English were ranked second-to-last and last, respectively (Dalton-Puffer et al. 1997: 121). Our point here is that L2 accent features are associated with social meanings: they are associated with less proficient speakers and are evaluated negatively.

To complement existing research with further empirical findings regarding the attitudes of L2 listeners toward variation in the frequencies with which L2 accent features are produced, we ran experiments on the perception and evaluation of /θ/ as [s] (e.g., *think* as [sɪŋk]), final devoicing of /d/ as [t] (e.g., *food* as [fuːt]), as well as a replacement of /w/ with [v] (e.g., *work* as [vɜːk]). Our choice of proficiency-based variation in consonant production was motivated by the fact that, unlike vowels, consonants are one of the most important cues to an L2 accent (Beinhoff 2013: 50). Based on the results of our experiments on (ing) and proficiency-based features, we can revisit and redefine the limits of sociolinguistic monitoring with regard to different types of frequency variation and their evaluation by L2 speakers of English – and make more detailed statements about the nature of sociolinguistic monitoring.

## 3 Current study

### 3.1 Methods

#### 3.1.1 Test passages

One of the main goals of this study was to revisit and re-evaluate existing assumptions about the workings of sociolinguistic monitoring in an environment in which English is mainly learned through L2 language instruction. With the aim of achieving a certain level of comparability with results generated in Labov et al.’s (2006, 2011) experiments, several sets of guises used in our study were based on an only slightly adapted version of the original news broadcast (see Appendix, Section 5.1.1 for a full list of adaptations). The first test passage, referred to here as the *Labov*-text, thus contained ten progressive {*-ing*} suffixes, with potential for realization with a velar [ɪŋ] and alveolar nasal [ɪn], respectively.

To address some shortcomings of the original newscast passage4Levon and Fox (2014, fn. 15), referring to a personal correspondence with Paul Foulkes, point out that the logarithmic response pattern found by Labov et al. could have resulted from the fact that ten (ing) tokens were spread across seven sentences only. Moreover, some tokens appeared in phonetic neutralization contexts, e.g., a velar plosive in the onset of the following word. We thus ensured that listeners were presented with only one token per headline item; all (ing) tokens were also followed by words beginning on a vowel. and to test the generalizability of claims about sociolinguistic monitoring to features that are not sociolinguistic in a strict sense, we additionally constructed our own test passage, referred to here as the *Headlines*-text. The *Headlines*-text not only allows us to establish distinctions between velar and alveolar realizations of (ing) across several guises. It was also designed to accommodate alternate pronunciations of word-initial /θ/ and word-final /d/, which have been shown to result from transfer phenomena in L2 phonology (see Major 2008). Accordingly, the new test passage also included ten tokens that allowed for replacing word-initial /θ/ with [s], so that a word such as *thought* would be pronounced [sɔːt] instead of [θɔːt]. Likewise, the text contained ten tokens that support assessing the effect of final devoicing on listeners’ perception and evaluations. Thus, words such as *food*, regularly pronounced as [fuːd], would be articulated as [fuːt]. In order to accommodate variation with regard to /w/ pronounced as [w] or [v], the *Headlines*-text had to be slightly adapted (see Appendix, Section 5.1.2 for a full list of adaptations). The variation we seek to represent with the respective sets of guises is thus proficiency-based (for (ths), (dt), (wv), but sociolinguistic for (ing)). Given that, in contexts of L2 teaching and learning, conforming to standard L1 features is the desired goal and diverging from the norm is regarded a production error, proficiency-based variation should give rise to evaluative responses.

To determine if evaluations may be affected by listeners’ (in)ability to *identify* the different variants of a variable, e.g., their ability to perceive word-final [d] and [t] as distinct variants, we conducted a small-scale ‘identification’ study with 15 L2 English listeners (from the same cohort as those who participated in the main experiments).5For each of the variables in focus here, i.e., (ing), (ths), final devoicing (dt), and (wv), we created three pairs of guises. Each pair was based on a short sentence (often drawn from the actual test passages, e.g., *Prices for food have been decreasing*.) and differed only with regard to the feature-specific variants (e.g., ([d]/[t])). Participants were first presented with two possible realizations of a variable (in writing, i.e., *In the following recording, is the word food pronounced as food [d] or as foot [t]?*). Then, they were asked to listen to a test sentence and to decide which variant they heard. Each participant listened to 12 sentences in total. Results show that listeners’ ability to identify distinct proficiency-based features decreases steadily from (ths) (93.3 % of tokens identified correctly) to (wv) (80.0 % correct responses) to (dt) (75.6 % correct responses). The differences in listeners’ ability to identify variants is similar to that of L1 speakers’ results for (ing), (x) (i.e., [x] for /k/) and (t)-deletion (see Schleef et al. Under review a), but needs to be kept in mind when interpreting response patterns for the professionalism ratings.

The *Headlines*-text also established a contrast in overall scope/length of the test passage. While the *Labov*-text consisted of 162 words, which were equivalent to 57 s of spoken text, the *Headlines*-text comprised 246 words, which took our speakers about 1 min 22 s to read out. The difference in length enables us to explore the “temporal window” (Labov et al. 2011: 457) in which listeners are able to monitor sociolinguistic variation for (ing) as well as proficiency-based variation.6We are aware of the fact that, in order to get more closely at the issue of temporal range, it is important to implement real-time methods as well (see e.g., Montgomery and Moore [2018]; Watson and Clark [2015] and the real-time study reported on in Labov et al. [2011: 454–457]). Such methods allow for an assessment of when exactly evaluations are formed and adapted over time instead of eliciting an overall reaction.


#### 3.1.2 Speaker profiles and guise construction

Test passages were recorded with two different speakers. Both speakers are female and in their mid-30s but differ with regard to their regional and linguistic background. Speaker 1 is an L1 speaker of Standard American English from Boulder, Colorado, USA. Speaker 2 is a highly proficient L2 speaker of English (level of proficiency approximates C2), who was born and raised in Carinthia, Austria; her L1 is (Austrian-)German. Both speakers were asked to read out both the *Labov*-text and the *Headlines*-text, first with a consistent pronunciation of (ing) as [ɪŋ] and a second time realizing all relevant tokens with the alveolar variant [ɪn]. In a similar fashion, the L2 speaker was also recorded reading the *Headlines*-text with varying realizations of word-initial /θ/, then with contrasting realizations of word-final /d/ and also with the different realizations of /w/. Speakers were recorded in a sound-attenuating booth at the University of Salzburg and were asked to read the texts multiple times until recordings approximated the style of a news broadcast; the best articulated versions were then selected and processed further to create the experimental stimuli.

Stimuli were then constructed by means of digital splicing in Praat (Boersma and Weenink 2022), following the procedure as described for the original experiment. In order to create the (ing) guises for both texts (*Labov* and *Headlines*) and speakers (L1 and L2), those recordings in which all (ing)-tokens were produced with [ɪŋ] constituted the 0 % guises. They also served as a basis for creating further versions of the respective recording with only one [ɪŋ], i.e., 10 %, replaced with [ɪn]. The 10 % guise, in turn, was edited further to change another [ɪŋ]-realization to [ɪn], thereby creating the 20 % guise. Following this method, four further guises were constructed per test passage and speaker, in which a total of three, five, seven or ten alveolar realizations were spliced into the base recording.7In order to ensure that our guises would be comparable to previous research (e.g., Levon and Fox 2014), we clarified the exact order of token splicing in a personal correspondence (April 2022) with Levon. Following Levon and Fox’s procedure, we first replaced token 5 (10 % guise), then also 7 (20 % guise), then 3 (30 % guise), then 9 and 1 (50 % guise), then tokens 6 and 4 (70 % guise), and finally tokens 2, 8, and 10 (100 % guise).


The procedure was repeated for the further L2-speaker recordings of *Headlines*, featuring contrasting articulations of word-initial /θ/, /w/, and word-final /d/, respectively.8As it seemed implausible to test L2 listeners’ evaluations of pronunciation features that are typical of the accents of German learners of English, we refrained from recording the L1 speaker reading the *Headlines*-text with varying articulation of (ths), (dt), and (wv). As with the (ing) guises, seven versions of the original recordings were created, with either 0 %, 10 %, 20 %, 30 %, 50 %, 70 % or 100 % of [θ]-, [d]-, or [w]-realizations cut and replaced with [s], [t], or [v], respectively.9We conducted a small experiment to test whether the insertion of the three L2 features into the original recording results in guises that are heard as *realistic* English spoken with a German accent. To do this, we created four guises based on the recordings used in the study that consisted of three sentences each. Three of these guises contained exactly three tokens of one of the three L2 features. We also created a guise based on the original recording with no manipulation at all. We recruited 20 participants within the target group of the study, who listened to the guises, preceded by a training guise. In each case, we asked: “How *realistic* is it that a(n Austrian) German L2 speaker of English would sound like this?” Participants answered on a seven-point rating scale that ranged from *perfectly realistic* (1) to *not realistic at all* (7). On average, all guises were rated as relatively realistic with means of 2.3 for the original guise, 2.6 for final devoicing (/d/ as [t]), 2.7 for /w/ as [v], and 2.8 for /θ/ as [s]. We conducted a mixed-effects linear regression with participant as random effect and the original guise as reference value and found none of the guises to be significantly different from the unmanipulated guise, which we assume to be realistic. The mixed-effects model is included in the Appendix, see Section 5.1.3, Table 5. Each set of guises showed variation only with regard to one variable. This means that variation in (ing), (ths), (dt), and (wv) never occurred in combination so that observed effects of guise on listener evaluations could be plausibly linked to one particular variable. In all cases, manipulations concerned the sounds in question. Only in very few cases did we have to replace small parts of the speech signal in the immediate phonetic environment. Table 1 provides an overview of the test passages, the speakers with whom the respective versions of the texts were recorded, and the variables in focus with the number of (valid) responses per guise set.

**Table 1:** Overview of test passages, speakers, and variables with number of (valid) responses per guise set. Total of 270 responses from 135 participants. For further information, see Appendix, Section 5.

Test passage	Speaker	Variable	No. responses
*Labov*-text	L1	(ing) [ɪŋ] / [ɪn]	41
	L2	(ing) [ɪŋ] / [ɪn]	39
*Headlines*-text	L1	(ing) [ɪŋ] / [ɪn]	33
	L2	(ing) [ɪŋ] / [ɪn]	51
*Headlines*-text	L2	(ths) [θ] / [s]	37
	L2	(dt) [d] / [t]	39
	L2	(wv) [w] / [v]	30

#### 3.1.3 Procedure and questionnaire

We set up a series of experimental sessions in which the stimuli were presented to participants. Each session included a combination of two sets of guises, with a 15-s pause between each guise and a 1-min pause in between sets. The order of guises within each set was randomized, except for the 50 % guise, which was always played first (see Labov et al. 2006, 2011; Levon and Fox 2014). Table 6 in the Appendix (Section 5.2) gives an overview of the sets of guises, the different orders in which guises were heard during experiments, as well as how the guise sets were combined.

Participants were told that they were going to hear several versions of two different trial newscasts that were meant to be submitted with a job application as a newscaster at an Austrian English-language radio station. They were instructed to listen to each recording in full and to rate it on a seven-point rating-scale ranging from *perfectly professional* (1) to *try some other line of work* (7) (see Labov et al. 2006, 2011; Levon and Fox 2014). This part of the questionnaire also included further questions on each of the two sets of recordings. These questions asked what listeners thought the speaker’s linguistic and regional background was. After the second guise set, we also inquired about participants’ own habits of pronouncing (ing). A third part was aimed at collecting further biographical information on the participants, e.g., gender, age, class background, highest educational qualification, current semester of studying, and time spent in an English-speaking country. It also asked participants to report the strand chosen for pronunciation classes as part of their English degree program (American or British English), and whether they had already passed a language-practice exam attesting C1-level proficiency in English.

#### 3.1.4 Participants

Experiments were conducted in June 2022 and February 2023 and involved 135 students across all experiments (101 identifying as *female*, 27 as *male*, and 7 identifying as neither female nor male). At the time of the study, all participants were enrolled in an English-studies course at the University of Salzburg. Almost all participants were L1 speakers of (Austrian-)German; one participant was an L1 speaker of Dutch with a high level of proficiency in German. At the time of the experiment, 61 % (*N* = 82) of the participating students had passed the C1 language-proficiency exam required for course progression in the Department of English and American Studies, and the other 39 % (*N* = 53) were yet to sit the exam.

#### 3.1.5 Data analysis

Responses were entered into an Excel spread sheet and processed further using R run in R Studio (R Core Team 2022). *Professionalism score* was treated as the dependent variable. *Guise*, i.e., the frequency of occurrence of [ɪn] (for /ɪŋ/), [s] (for /θ/), [t] (for /d/, final devoicing), and [v] (for /w/), was treated as an independent variable. Further independent variables were participants’ (1) *gender* and (2) *age*, (3) the *strand* chosen for pronunciation classes (either American or British English), (4) whether or not students had passed the *C1 exam*, (5) whether or not students had spent time in an *English-speaking country*, (6) whether or not participants reported *using* [ɪn] *themselves*, and (7) whether participants believed they heard an *L1- or L2-speaking newscaster*. Although the effect of these predictors was checked, only those that improved the model fit were included for analysis in the final models. In all mixed-effects regression models, *Respondent* was entered as random effect. Mixed-effects models were built with the lmer() function available in the lmerTest package (Kuznetsova et al. 2017). Model fit checks were undertaken through the check_model() function from the performance package (Lüdecke et al. 2021) and the report package (Makowski et al. 2021).

### 3.2 Results

#### 3.2.1 Sociolinguistic variation

##### [ɪn] for /ɪŋ/

**Figure 1:**
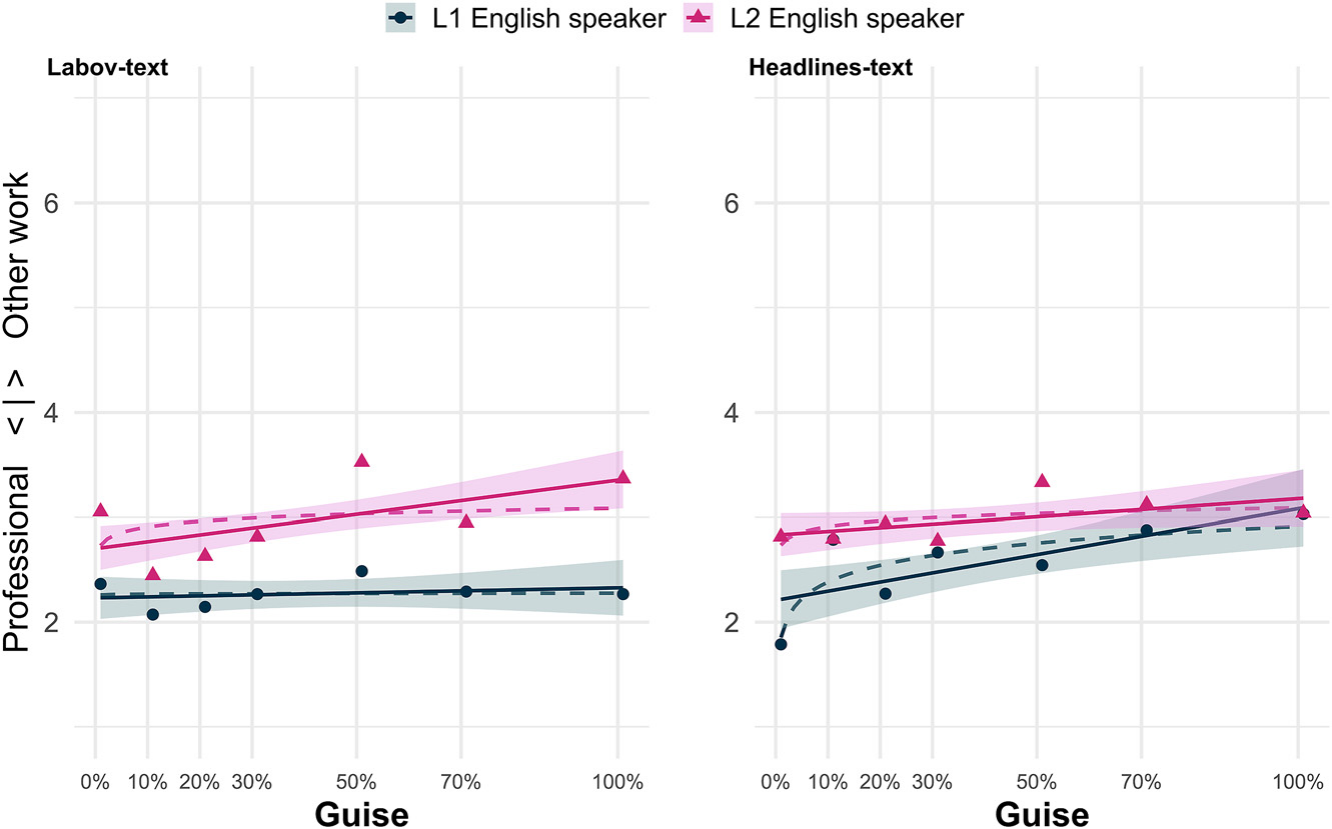
Results of professionalism score plotted against (ing) guise for (1) the *Labov*-text (*N* = 80), and (2) the *Headlines*-text (*N* = 84, cf. Table 1). Dashed lines represent the logarithmic progression fit and the unbroken lines represent the linear model fit to the data; mean score per guise is represented by the triangles and dots respectively.

Turning first to the *Labov*-text (see Table 2), we find no significant main effect for (ing) guise (*Est.* = 0.001, *p* = 0.53), in line with findings in Levon and Fox (2014) for evaluations of manipulated (ing) guises. However, looking at the variation between newscasters in general, irrespective of which particular guise was evaluated, listeners rated the L2 newscaster significantly lower on the professionalism scale than they rated the L1 speaker (*Est.* = 0.48, *p* = 0.01). While there was no main effect for guise, the significant interaction term between (ing) guise and L2 speaker (*Est.* = 0.01, *p* < 0.01) shows that respondents did rate an increase in [ɪn]-frequencies as less professional. As can be seen in Figure 1, the scores for neither the L1 newscaster nor the L2 newscaster follow a logarithmic pattern, and the logarithmic smooth line barely deviates from the linear fit line for either newscaster. Evaluations for both the L1 and the L2 newscaster are linear and fairly flat, although the positive but non-significant relationship between guise percentage and lower professionalism score can be seen for the L2 speaker. While there was no significant difference in the responses of those who self reported using [ɪn] for /ɪŋ/ themselves and those who did not (*Est.* = 0.21, *p* = 0.28), including this predictor was found to improve the overall model fit.

**Table 2:** Results of the mixed-effects models predicting score on the professionalism scale by (ing) guise for: (1) the *Labov*-text (*N* = 80), and the *Headlines*-text (*N* = 84).

	Estimate	Std. error	t-Value	p-Value	

*Labov*-text (ing)					
(Intercept)	2.06	0.21	9.85	<0.001	***
Guise	0.001	0.002	0.63	0.53	
Newscaster = L2	0.48	0.19	2.48	0.01	*
Do you use [ɪn] = yes	0.21	0.19	1.07	0.28	
Guise: L2 speaker	0.01	0.002	2.71	<0.001	**

***Headlines*-text (ing)**					

(Intercept)	2.34	0.16	14.28	< 0.001	***
Guise	0.005	0.001	4.46	< 0.001	***
Newscaster = L2	0.40	0.20	2.01	0.05	.

We make similar findings when looking at the *Headlines*-text results. Again, the L2 newscaster is rated as less professional sounding than the L1 newscaster (*Est.* = 0.40, *p* = 0.05). In the *Headlines*-text there was also a significant main effect for guise (*Est.* < 0.01, *p* < 0.01), whether or not respondents heard guises from the L1 or L2 speaking newscaster, they rated guises with more [ɪn] for /ɪŋ/ tokens as less professional sounding.

#### 3.2.2 Proficiency-based variation

##### [s] for /θ/

**Figure 2:**
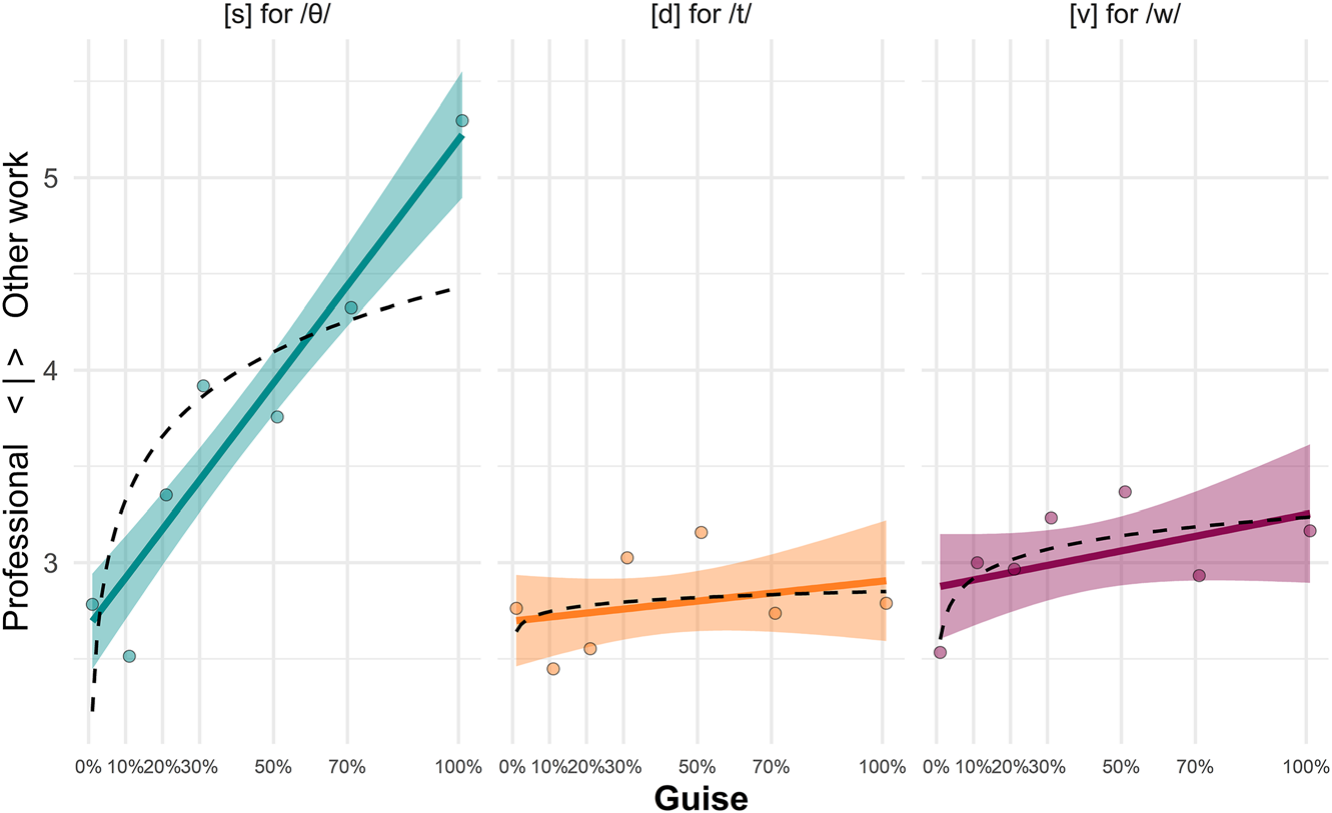
Scores on the professionalism scale for each of the three proficiency-based variants commonly found in German L2 speakers of English: (1) /θ/ realized as [s] (*N* = 37), (2) /d/ realized as [t] (*N* = 39), and (3) /w/ realized as [v] (*N* = 30) (cf. Table 1 for participant numbers).

Turning to the first of the proficiency-based features (in which respondents heard only the L2 English newscaster), the [s] for /θ/ guises are found to elicit significantly lower professionalism ratings as [s] tokens increase in number (*Est.* = 0.03, *p* < 0.001). As can be seen in Table 3 and Figure 2, the [s] for /θ/ guises attract some of the lowest overall professionalism ratings as [s]-percentage increases. Although the linear progression is found to be a better model fit than the logarithmic model (see Table 4), we can see for the first time in this data a distribution which starts to approximate the logarithmic fit postulated by Labov et al. (2006, 2011).

**Table 3:** Results of the mixed-effects models predicting score on the professionalism scale by guise for the proficiency-based variants: (1) /θ/ realized as [s], (2) /d/ realized as [t], and (3) /w/ realized as [v].

	Estimate	Std. error	t-Value	p-Value	

*Headlines*-text (ths)					
(Intercept)	2.70	0.17	16.22	<0.001	***
Guise	0.03	0.002	13.58	<0.001	***

***Headlines*-text (dt)**					

(Intercept)	2.85	0.18	15.69	<0.001	***
Guise	0.002	0.001	1.41	0.16	
lived abroad = yes	−0.83	0.40	−2.08	0.04	*

***Headlines*-text (wv)**					

(Intercept)	2.88	0.19	15.25	<0.001	***
Guise	0.004	0.002	1.99	0.05	.

**Table 4:** *r* ^2^ and AIC values for linear and log-transformed model fit comparisons. LB = *Labov*-text. HL = *Headlines*-text.

Feature	Model	*r* ^2^	AIC	*χ* ^2^
LB (ing)	Linear	0.46	1,543	***
	Log	0.44	1,555	
HL (ing)	Linear	0.43	1765	
	Log	0.44	1762	
HL (ths)	Linear	0.58	801	***
	Log	0.48	853	
HL (dt)	Linear	0.60	727	
	Log	0.60	728	
HL (wv)	Linear	0.49	621	
	Log	0.46	615	

##### [t] for /d/

Professionalism ratings were not found to be significantly affected by guise for this feature, meaning that respondents did not overall rate the newscaster as sounding less professional as she used more [t] for /d/ tokens. Although non-significant, the trend in the data does follow the same direction as the other features,10There is however a significant main effect for time spent abroad, with those who report having spent time living in an English-speaking country (e.g., as part of the Erasmus scheme or year abroad) rating this feature as significantly more professional-sounding than those who have not spent time in an English-speaking country (*Est*. = -0.83, *p* = 0.04). with evaluations moving toward less professional as [t]-percentage increases (*Est.* = 0.002, *p* = 0.16).

##### [v] for /w/

For the final proficiency-based feature, increasing the number of tokens of [v] for /w/ (e.g., [v]*eather* for /w/*eather*) does seem to elicit significantly lower ratings on the professionalism scale from the German L1 listeners in our sample (*Est.* = 0.004, *p* = 0.05). This finding is, however, right on the edge of what we would accept as significant in this study (*α* < 0.05).

#### 3.2.3 Logarithmic model fits

A summary of models can be seen in Table 4. All models were run on both linear and logarithmic transformed data to test whether there was any evidence in the data for the logarithmic distribution of evaluations found by Labov et al. (2006, 2011). Model fit was first assessed by means of (i) *r* ^2^ (the higher the *r* ^2^, the higher the amount of variation in the data the model is able to explain) and (ii) the AIC (the lower the AIC, the better the model fit).

In terms of the *r* ^2^ values, whether the data was log-transformed or not made very little difference to the overall model fit. In the *Headlines*-text (ing) guises, the log model was able to explain around 1 % more of the variation in the data than the linear model. In general, however, the linear models performed better, or very similarly to the log models. Looking at the AIC values, there was also very little difference between the two types of models for each feature. The *Labov*-text (ing) guise and the [s] for /θ/ guise (*Headlines*-text) both performed better as linear models than their log-transformed counterparts, but for all the other features, ANOVA testing revealed no significant difference between model fits.

## 4 Discussion and conclusion

Previous research on sociolinguistic monitoring has begun to shed light on the relationships between listeners’ evaluations of quantitative differences in linguistic variation on the one hand, and listener/speaker attributes (e.g., regional background, gender/sex, pragmatic language ability) or linguistic properties (e.g., perceptual salience, phonological context, structural level, word frequency/typicality) on the other. Based on such empirical work, several properties of sociolinguistic monitoring have been proposed, for instance, regarding its sensitivity, temporal and evaluative range. However, the question of whether L2 English speakers show response patterns similar to those of L1 speakers still lacked sufficient empirical grounding. We thus replicated Labov et al.’s study in a context in which English is acquired as a second (or other) language. A further goal of the present study was to test whether the workings of sociolinguistic monitoring can be generalized to cases of proficiency-based variation. Results from this perception study with 135 L2 English listeners support that sociolinguistic monitoring is not limited to classic sociolinguistic variation but extends to other cases of variation as well. For this reason, the process should be more aptly called *monitoring of socially meaningful variation*. Our results are suitable for various hypothesized ideas about monitoring – be that as a distinct cognitive module or variation monitoring as part of a more general capacity of human cognition.

### 4.1 Properties of sociolinguistic monitoring: evidence from L2 English listeners

Based on the response patterns for sociolinguistic variation of (ing) in the *Labov*-text and the *Headlines*-text, we are able to substantiate and refine core assumptions about the nature of sociolinguistic monitoring.

– **Temporal range**: Guise was found to predict listener evaluations of (ing)-based variation in both versions of the *Headlines*-text. In other words, L2 listeners seem generally capable of tracking sociolinguistic variation up to and over 1 min 22 s. Consequently, the ‘temporal window’ in which sociolinguistic monitoring takes place may be notably longer than 57 s, i.e., the length of the original text used by Labov et al. (2006, 2011).11A reviewer suggested that, since respondents were asked to log their rating only after the guise had played through, their evaluations might refer to a ‘unit of presentation’ rather than provide evidence of an extended temporal range of monitoring. We agree that this is a plausible interpretation but believe that, even if evaluations refer to the recording as a whole, its *scope* may still be relevant to listeners’ evaluations. The finding that evaluations of (ing)-based variation in the *Labov*-text merely indicated a non-significant effect of L2 newscaster may result from the size of the data sample. Generally, we must be careful when evaluating negative results, which may become significant with more data. The fact that the effect is more likely to be evident in the L2-newscaster guises may also be due to a more general negative bias toward L2-accented English and, hence, stricter assessment (see Dalton-Puffer et al. 1997).
– **Attenuation**: Regarding attenuation, we are able to further substantiate previous claims that some data can be better described by linear rather than logarithmic model fits. The ceiling effect reported by Labov et al. (2006, 2011) was generally not found in our data. We can thus conclude that many listeners process noticeable non-standard features in a linear rather than a logarithmic fashion. This means that each token results in the same effect increase, rather than consecutive tokens having a reduced effect.
– **Gender/sex**: As in previous studies, our results do not point to gender/sex as a predictor of professionalism ratings by L2 listeners.
– **Age of acquisition**: Our data does not indicate an age-effect either, although participants were not distributed widely enough across different age groups for us to be able to make valid statements about age of acquisition as a factor. Proficiency also did not matter at a statistically significant level regarding B2, C1, and C2 listeners. It is unlikely that listeners with proficiency levels below B2 would even be able to complete the experimental task in a meaningful way.

### 4.2 Generalizability of properties to proficiency-based variation

Regarding the question of how *specialized* the phenomenon of sociolinguistic monitoring is, we found clear evidence that the tracking and evaluation of quantitative linguistic variation is not limited to instances of sociolinguistic variation alone. There is much evidence that it ought to be regarded a more general phenomenon of social perception and evaluation glossed *monitoring of socially meaningful variation*. Response patterns to the *Headlines*-test passages indicate that (Austrian-)German learners of English are equally capable of tracking quantitative differences in proficiency-based variation over a period of more than 80 s. Moreover, L2 listeners attributed social meanings to quantitative differences in proficiency-based variation. In other words, they seem to have an understanding of what counts as professional in the context of English-language news reporting in Austria. However, not all L2-accent features were tracked and evaluated equally. While L2 listeners showed sensitivity to variation in (ths) and (wv) in the *Headlines*-text with each token having the same effect (linear model fit), evaluations of variation in final devoicing (dt) (*Headlines*-text) were unaffected by guise.

These results can now be interpreted against the backdrop of the small-scale ‘identification’ study, which complemented the main experiments. As explained in further detail in Section 3.1, listeners’ ability to identify variants differed depending on the variable in question. Among the proficiency-based variables, variation in (dt) proved the most difficult to discriminate. The fact that evaluations of (dt)-based variation were unaffected by guise could thus at least partly result from L2 listeners’ difficulties in hearing word-final [d] and [t] as distinct variants. Moreover, results also suggest that (ths)-variants, of all the L2-accent features tested, were the easiest to discriminate. We can then assume that the significant effect of (ths)-based variation on evaluations results at least partly from L2 listeners’ strong ability to *hear* [θ] and [s] as distinct variants.12Naturally, more data is needed to verify the observed differences in listeners’ ability to identify variants. If we then subscribe to the view that identifying variants with ease is a ‘favoring condition’ of a variable’s perceptual salience overall (see Llamas et al.’s [2016] overview of research into language-external and -internal factors contributing to perceptual salience), we can furthermore hypothesize that (ths)-based variation is regarded by German learners of English as a strong L2-accent feature.13It needs to be kept in mind, however, that a listener’s ability to identify variants is not a necessary precondition of salience. Variables, such as (th)-fronting, tend to be relatively hard to discriminate acoustically (see Miller and Nicely 1955: 347, qtd. in Llamas et al.’s 2016: 3). Nevertheless, it has been shown to bear much social salience in particular regional contexts (see Levon and Fox 2014). Due to a widespread stigmatization of accent features in the work domain (see Ramjattan 2023), it may then act as a marker of decreased professionalism and is likely to prompt negative evaluations. This could explain why, in our experiments, the (ths)-guises received the lowest professionalism ratings overall. As with the results for (ing)-based variation, evaluations of proficiency-based variation seem to be independent from gender and age differences between respondents.

### 4.3 Implications for future research

Based on the results of our study, several implications for future research can be identified. Firstly, further work is necessary to explain why exactly, in some cases, professionalism ratings were entirely independent from frequency differences in both sociolinguistic and proficiency-based variation. Here, further potential effects such as a negative bias toward L1- versus L2-accented English or the question of whether listeners are able to hear different variants as distinct would need to be considered in further detail. Indeed, listeners’ ability to identify variants seems to be one of the most pressing concerns – not just for L2 listeners but also for L1 listeners. Clearly, findings from monitoring experiments are not conclusive evidence that listeners are or are not able to discriminate certain variants in perception. Also, as mentioned above, the ability to identify variants is not a precondition of social salience *per se*. However, such results provide an important backdrop against which attitudinal patterns can be interpreted. In the future, research into the social meanings of linguistic variation should be complemented with an investigation of listeners’ ability to identify, notice, and discriminate the variants of a variable (see Schleef et al. Under review b).

Secondly, future research should explore further potential reasons as to why certain variables lead to a linear distribution of evaluations whereas others are associated with a logarithmic one. Further insights can be gleaned by testing further (types of) variables (e.g., discourse markers) and through comparison with real-time response data. In any case, as Levon and Fox (2014) (and others, see above) have also shown, logarithmic model fits should not be considered a defining criterion of the tracking and evaluation mechanisms that are part of sociolinguistic monitoring.

Finally, regarding the spectrum of social evaluations, there are still many more questions than there are answers. Our study set a focus on ratings regarding professionalism. However, incorporating further scales, e.g., trustworthiness or likability, would complement existing research and enable us to paint a more complex picture of L2 listeners’ attitudes toward quantitative differences in sociolinguistic and proficiency-based variation.

## 5 Appendix

### 5.1 Test passages

#### 5.1.1 Adapted *Labov*-text

The following test passage, which was used here to test the evaluation of (ing)-based variation, is based on Labov et al.’s (2006, 2011) original text. In the context of our study, however, some slight adaptations were considered necessary. In several places, Labov et al.’s text included references to news actors, e.g., *President Bush*, *Senator Edward Kennedy*, or *Federal Reserve Board chairman Alan Greenspan*. In regular news discourse, such references add to the *newsworthiness* of a text (see Bednarek and Caple 2017) and thus make the constructed passage more authentic to study participants. In order to retain the text’s authenticity in our context, we rephrased any references to real-world politicians in a more generic fashion, e.g., replacing the proper nouns given above with *The President*, *Senator Kennedy*, and *The Federal Reserve Board chairman*.

– The President announced tonight that he was **putting** ([ɪŋ]/[ɪn]) all available White House resources into support for the new tax cut bill.
– Democratic leaders of the House and Senate are **preparing** ([ɪŋ]/[ɪn]) compromise legislation.
– Republican spokespersons predicted that record numbers of **working**-class ([ɪŋ]/[ɪn]) Americans would be **receiving** ([ɪŋ]/[ɪn]) tax refund checks before the end of the year.
– Senator Kennedy’s staff announced that the tax cuts are **creating** ([ɪŋ]/[ɪn]) a new elite who are excused from **paying** ([ɪŋ]/[ɪn]) their fair share of the cost of government.
– At the Office of Management of the Budget, officials are **trying** ([ɪŋ]/[ɪn]) to estimate the size of the deficit that will be produced by the new legislation.
– The Federal Reserve Board chairman stated that he was not **confirming** ([ɪŋ]/[ɪn]) that tax cuts would lead to a change in prime interest rates, nor was he **denying** ([ɪŋ]/[ɪn]) it.
– The Washington Post is **publishing** ([ɪŋ]/[ɪn]) today a list of all members of Congress, who will receive tax refunds greater than $1,000 as a result of the proposed tax cuts.

#### 5.1.2 *Headlines*-text

The *Headlines*-test passage was meant to accommodate instances of both sociolinguistic (ing) and proficiency-based variation (dt, ths). Below, all potential instances of variation are indicated in the same text; the experiments, however, always focused on one variable per guise set.

Our top stories:

– The government denies rumors of a tax rise after a **flood** ([d]/[t]) of tweets about the hike began **spreading** ([ɪŋ]/[ɪn]) online. A Twitter typo **thought** ([θ]/[s]) to be the origin of the confusion went viral yesterday afternoon.
– The new CEO of a local **wood** ([d]/[t]) and metalworks company was seen **meeting** ([ɪŋ]/[ɪn]) a group of close to **thirteen** ([θ]/[s]) hundred furious workers who have now gone without pay for ten weeks.
– The government came under fire yesterday from the **head** ([d]/[t]) of the workers’ union for **putting** ([ɪŋ]/[ɪn]) over a **thousand** ([θ]/[s]) jobs in danger.
– A recent study shows that the **mood** ([d]/[t]) of the nation has been **improving** ([ɪŋ]/[ɪn]) over the last year partly **thanks** ([θ]/[s]) to a successful economy.
– Inflation has fallen slightly. Prices for **food** ([d]/[t]) as well as other essentials have been **increasing** ([ɪŋ]/[ɪn]) after a **thorough** ([θ]/[s]) review of import tariffs.
– Numbers on hourly income are **good** ([d]/[t]) across the area, actually **rising** ([ɪŋ]/[ɪn]) in most sectors, except for the Arts where many **theatres** ([θ]/[s]) have seen a decline in numbers.
– A consumer organization, which specializes in identity **fraud** ([d]/[t]), is **urging** ([ɪŋ]/[ɪn]) all shoppers to be mindful of identity **theft** ([θ]/[s]), especially online.
– Senior citizens in **need** ([d]/[t]) of government support can now get advice from a new Senior Citizens Helpline, which is **providing** ([ɪŋ]/[ɪn]) a list of new **therapy** ([θ]/[s]) options.
– Scientists develop a new digital tool to increase the **speed** ([d]/[t]) in **collecting** ([ɪŋ]/[ɪn]), on a large scale, the **thoughts** ([θ]/[s]) and the opinions of the public.
– The weather will remain pretty **bad** ([d]/[t]) all week. We are **expecting** ([ɪŋ]/[ɪn]) afternoon temperatures to drop by **thirty** ([θ]/[s]) degrees.

In order to accommodate proficiency-based variation with regard to (wv), i.e., /w/ pronounced as [w] or [v], the *Headlines*-text had to be slightly adapted. In order to allow for controlled variation, we replaced any further words with potential for (wv)-variation beyond those ten tokens we sought to manipulate in guise production. Places in which the (wv)-version diverges from the other versions of the *Headlines*-text are indicated as follows: (text in previous version ➤) new text.

Our top stories:

– The government denies rumors of a tax rise after a flood of tweets about the hike began spreading online. A Twitter typo, thought to be the origin of the confusion, **was** ([w]/[v]) (went viral ➤) shared extensively yesterday afternoon.
– The new CEO of a local **wood** ([w]/[v]) and metalworks company (was seen ➤) is meeting a group of close to thirteen hundred furious (workers ➤) employees, who have now had no pay for (ten weeks ➤) two months.
– The government came under fire yesterday from the head of the **workers’** ([w]/[v]) union for putting over a thousand jobs in danger.
– A recent study shows that the mood of the nation has been improving over the last (year ➤) **weeks** ([w]/[v]) partly thanks to a successful economy.
– Inflation has fallen slightly. Prices for food as **well** ([w]/[v]) as other essentials have been decreasing after a thorough review of import tariffs.
– Numbers on hourly income are good across the area, actually rising in most sectors, except for the Arts **where** ([w]/[v]) many theatres have seen a decline in numbers.
– A consumer organization, **which** ([w]/[v]) specializes in identity fraud, is urging all shoppers to be mindful of identity theft, especially online.
– Senior citizens in need of government support can now get advice from a new Senior Citizens Helpline that is providing a list of (new therapy ➤) **wellness** ([w]/[v]) and therapy options.
– Scientists develop a new digital (tool ➤) **web**-tool ([w]/[v]) to increase the speed in collecting, on a large scale, the thoughts and the opinions of the public.
– The **weather** ([w]/[v]) (will remain ➤) remains pretty bad all (week ➤) day. You should be expecting afternoon temperatures (to drop by thirty degrees ➤) in the low thirties.

#### 5.1.3 Naturalness of the *Headlines*-guises with L2 features

**Table 5:** Results of the mixed-effects model showing ratings of how realistic the audio-recordings of each variable were rated, with the neutral guise as the reference level.

	Estimate	Std. error	df	t-Value	p-Value	
(Intercept)	2.61	0.41	41.15	6.32	<0.001	***
Feature = ing	−0.11	0.36	39.84	−0.31	0.76	
Feature = ths	−0.15	0.54	47.35	−0.29	0.78	
Feature = dt	−0.40	0.54	47.35	−0.75	0.46	
Feature = wv	0.42	0.37	35.66	1.12	0.27	

### 5.2 Combination of guise sets and guise orders

Table 6 gives an overview of the combinations in which guise sets were heard during experiments. Whenever a combination included a guise set recorded with the L2 speaker, it was this set that the experiment began with. This was done in response to results from a previous study by Dalton-Puffer et al., which found that L2 English learners from Austria showed clear preference for L1 standard pronunciation when rating L1- and L2-accented versions of a radio announcement (Dalton-Puffer et al. 1997: 125). By playing the L1-accented sets of guises second, whenever they were combined with L2 speaker recordings, we sought to avoid any evaluative bias. Table 6 also details in which order individual guises were played. Except for the 50 % guise, which was always heard first, guises appeared in random order (see Labov et al. 2006, 2011; Levon and Fox 2014).

**Table 6:** Comb = Combination of guise sets; Txt = Test passage used; LB = *Labov*-text; HL = *Headlines*-text; Var = Variable manipulated to create the seven guises that make up a guise set; Sp = Speaker; Order = Guise order; Squ = Specification of ‘guise order’, i.e., actual sequence in which guises appeared in the experiment.

Comb	Txt	Var	Sp	Ord	Squ						
1	HL	ing	L2	A	50	0	100	70	20	30	10
	LB	ing	L1	B	50	20	0	10	30	70	100
2	HL	ing	L2	C	50	20	100	10	70	30	0
	LB	ing	L1	D	50	70	0	30	10	20	100
3	LB	ing	L2	E	50	100	0	70	30	20	10
	HL	dt	L2	F	50	30	10	0	70	100	20
4	LB	ing	L2	G	50	30	100	70	10	0	20
	HL	dt	L2	H	50	0	70	100	20	30	10
5	HL	ths	L2	I	50	20	0	70	30	10	100
	HL	ing	L1	J	50	100	20	10	30	70	0
6	HL	ths	L2	K	50	70	0	20	30	100	10
	HL	ing	L1	L	50	20	10	100	70	0	30
7	HL	ing	L2	M	50	70	20	100	30	10	0
	HL	ths	L2	N	50	10	0	20	30	70	100
8	HL	wv	L2	O	50	10	100	30	70	0	20
	LB	ing	L1	P	50	70	30	0	20	100	10
9	HL	ing	L2	Q	50	10	70	0	100	30	20
	HL	wv	L2	R	50	30	70	100	0	20	10
10	HL	wv	L2	R	50	30	70	100	0	20	10
	HL	ing	L1	T	50	70	30	10	100	0	20
11	HL	ths	L2	I	50	20	0	70	30	10	100
	LB	ing	L1	B	50	20	0	10	30	70	100
